# Does Co-worker Presenteeism Increase Innovative Behavior? Evidence From IT Professionals Under the 996 Work Regime in China

**DOI:** 10.3389/fpsyg.2021.681505

**Published:** 2021-07-01

**Authors:** Tianan Yang, Ran Liu, Jianwei Deng

**Affiliations:** ^1^School of Management and Economics, Beijing Institute of Technology, Beijing, China; ^2^Sustainable Development Research Institute for Economy and Society of Beijing, Beijing Institute of Technology, Beijing, China

**Keywords:** co-worker presenteeism, promotion focus, innovative behavior, IT professionals, 996 work regime

## Abstract

Drawing on the event system and regulatory focus theory, this study constructed an impact mechanism model to investigate the relationship between the event strength of co-worker presenteeism and innovative behavior among IT professionals under the 996 work regime. In addition to test the direct effect, we examined the indirect effect of promotion focus and the moderating effect of event time in this relationship. Data were collected through an online survey administered to 374 IT professionals in China. The results showed a positive relationship between the criticality of co-worker presenteeism events and innovative behavior. An indirect effect of promotion focus was also found in this relationship. The timing of co-worker presenteeism events moderated the relationship between the criticality of co-worker presenteeism events and promotion focus. Specifically, the effect was more significant when co-worker presenteeism events occurred during project delays.

## Introduction

Although the negative effects of presenteeism have been well researched, the positive consequences have received relatively less attention, and the effects of presenteeism on co-workers are largely unknown. Co-worker presenteeism refers to co-workers attending work despite being in a state of suboptimal health (Johns, 2010; Karanika-Murray and Biron, 2019; Ruhle et al., 2020). The COVID-19 pandemic increased employees’ levels of uncertainty about their job characteristics and work context (Tang et al., 2020). In a large-scale survey of US employees, 96% of the participants reported that the pandemic had affected their stress levels and considered it the most stressful period of their professional careers (Ginger, 2020), with stress being an established factor in poor psychological and physical health (Zurlo et al., 2016). The pandemic has also spurred the rapid uptake of digital communication, services and consumption (e.g., telecommuting, online healthcare, online education and online fresh food shopping), which is demanding higher levels of efficiency and innovation from employees of related IT enterprises. With these developments in the IT industry and the associated increases in occupational stress, the phenomenon of co-worker presenteeism among IT professionals has sharply increased during the period of the COVID-19 pandemic against the background of the already demanding ‘996’ work regime.

The sudden death of a 23-year-old employee of the Pinduoduo e-commerce company on 29 December 2020 triggered heated discussions in China surrounding the 996 work regime (Damaojingjing, 2020). The 996 regime refers to a widespread regulation among IT companies in China that employees work from 9 a.m. to 9 p.m., 6 days a week. The 996 work regime has become a default corporate culture in IT companies over recent years and is sometimes mandatory. The rule has been adopted by many other well-known Chinese IT companies, such as Alibaba, Tencent and Jingdong (Xiao et al., 2020). The term originated from a project called ‘996ICU’, which was uploaded to GitHub by a software programmer on 27 March 2019 as an act of protest. The project listed the companies requiring a 996 working pattern for blacklisting and promoted the slogan ‘developers’ lives matter’ (Yang, 2019). Nevertheless, Jack Ma, the influential founder of Alibaba, expressed his support for 996 on his official Weibo account on 12 April 2019.

With almost all enterprises now facing a dynamic environment, organizations are reliant on innovation to survive and to gain competitive advantages (Han and Yang, 2011; Anderson et al., 2014). This is especially true for digital ventures and software development companies during and in the aftermath of the COVID-19 pandemic, as they must adapt to a rapidly changing market through innovation aimed at developing high-quality products and providing excellent services (Huang et al., 2017; Kude et al., 2019). Individual creativity is the foundation of an organization’s innovation (Amabile, 1988), and for IT enterprises, as typical examples of knowledge-based organizations, innovative behavior by their professional employees is the primary source of their competitiveness.

In meeting the innovation needs of enterprises, employees naturally face the problem of pacing their work, and an organization’s regulation of the intensity and speed with which its members operate is crucial to innovation management (Gersick, 1994; Dougherty et al., 2013). The 996 work regime is a typical manifestation of the time-pacing regulations of IT companies, especially against the background of the COVID-19 pandemic. It is not surprising to find that presenteeism, meaning to work in a state of suboptimal health, is commonly reported by IT professionals (Karanika-Murray and Biron, 2019). The unique working environment of IT professionals, which is often characterized by high perceived workloads, role ambiguity and role conflict, can easily induce both work exhaustion and presenteeism (Demerouti et al., 2009; Shih et al., 2013). The working patterns of IT professionals might mean that presenteeism is particularly prevalent in the IT industry.

In the past two decades, most studies on the impact of presenteeism believe that it is a kind of negative behavior or it has negative effects for organizations, teams, or individuals. However, a small but growing body of the literature is turning to explore the positive side of it. The direction expansion of the positive effects research can be understood deeply from the conceptual connotation and practical observation of presenteeism (Karanika-Murray and Biron, 2019).

First, there are two main definitions of presenteeism: one that emphasizes the act of working while ill and the other that focuses on the loss of productivity due to poor health conditions (Johns, 2010; Lohaus and Habermann, 2019). However, by either definition, the nature of presenteeism phenomenon can be partially understood if it focuses only on health-related issues and ignores the importance of work itself (Karanika-Murray and Biron, 2019). Presenteeism is also considered as an adaptive behavior that serves the purpose of balancing health constraints and job performance requirements, rather than just a negative behavior (Karanika-Murray and Biron, 2019). Second, from the observation of the real situation, employees can participate in work when their health conditions are not serious, and participation in work can help people meet some basic psychological needs, keep job control and maintain working relationship with colleagues and clients, which is conducive to recovery from illness to a certain extent (Demerouti et al., 2009; Van den Broeck et al., 2016; Ruhle et al., 2020). A growing body of evidence shows that presenteeism has certain positive effects on both individuals and organizations.

Therefore, based on the conceptual connotation and realistic research evidences, we should not solely focus on the negative effects of presenteeism, but should try to explore the positive aspects of it.

Although there has been extensive research undertaken on the outcomes of presenteeism, four aspects of the phenomenon are worthy of further exploration, especially against the background of the 996 work regime and in the aftermath of the COVID-19 pandemic. First, most studies have explored the negative effects of presenteeism, arguing that it is bad for the productivity of organizations and individuals. Studies focusing on the positive effects of presenteeism are relatively fewer but are increasing in number. A few studies have argued that presenteeism is an example of adaptive or organizational citizenship behavior (Miraglia and Johns, 2016; Karanika-Murray and Biron, 2019) with benefits for individual innovation performance (Xu et al., 2016). The present study enriches this body of research into the positive effects of presenteeism. Second, most studies have focused on the effects of presenteeism on the individual, with few having explored interpersonal effects, such as whether and how presenteeism affects the behavior of other employees (Luksyte et al., 2015). Grounded in event system theory (EST; Morgeson et al., 2015), the present article focuses on the effects of co-worker presenteeism on innovative behavior from an interpersonal perspective. Third, studies of the mediation mechanism between co-worker presenteeism and employee output have mostly adopted the perspective of discrete emotional responses (Luksyte et al., 2015), which are relatively situational and transient. However, the behavior of colleagues can also stimulate responses from some relatively stable traits, such as individual self-regulation preferences. According to EST and regulatory focus theory (RFT; Higgins, 1997), each person has a different regulatory focus for coping and responds differently to events occurring at different times. This paper expands on the research into the mediation mechanism between co-worker presenteeism and employee output from the perspective of self-regulation and, based on EST, further explores the boundary condition of event time on the relationship between colleague presenteeism events and individual regulatory focus. Fourth, most research on the antecedents of innovative behavior, such as the attributes of the work, individual personality traits, or such situational factors as leadership style and organizational climate, has focused on the stable characteristics of the entities and has rarely explored the event-related antecedents. The present study extends this research into innovative behavior antecedents by considering event-related factors.

To fill these research gaps, this study draws on a sample of IT professionals to build an impact mechanism model of the relationship between co-worker presenteeism and employees’ innovative behavior based on the EST (Morgeson et al., 2015) and RFT (Higgins, 1997). This study addresses the following research questions (1) Does co-worker presenteeism event strength affect innovative behavior among IT professionals in the context of the 996 work regime? (2) What is the regulatory focus-related mediation mechanism between co-worker presenteeism and innovative behavior among IT professionals? (3) What is the boundary condition in the relationship between co-worker presenteeism and individual regulatory focus?

## Theory and Hypotheses

### Theoretical Background

#### Event System Theory

The main paradigm of management research involves attending to the stable characteristics of the entity under study, which has meant that there has been relatively little research into the potentially transformational effects of events experienced by an entity (Liu and Liu, 2017). In contrast, EST systematically considers the different attributes of an event and its mechanism of influence on the entity. EST predicts that event strength (generated by criticality, novelty, etc.), event time (including timing and duration) and event space (including origin and spatial dispersion) affect the entity individually or collectively and directly or indirectly.

The EST points out that the attributes of strength, time and space of an event determine the influence degree of an event on an entity. For criticality in event strength, it reflects the extent to which the event requires priority response by the organization, and has a significant impact on the realization of the organization’s goals (Liu and Liu, 2017). The more critical the event is, the more attention it requires the organization to pay. For instance, a more critical event is considered more likely to influence or trigger behaviors, characteristics and new events. Event time is posited as a moderator in the relationship between the event strength and the outcomes. Furthermore, events that are more consistent with the development stage of the entity are more influential (Morgeson et al., 2015). In addition, Liu and Liu (2017) pointed out that it is often difficult for researchers to study the three attributes of an event (strength, time and space) simultaneously; therefore, scholars ought to consider one or two of these attributes in combination with their own research focus to predict the corresponding dependent variables.

In our research model, we regard colleague presenteeism as an event, explore the influence path and mechanism of co-worker presenteeism event strength (criticality) on employees’ innovative work behavior and combine the RFT to explore the moderating effect of event time (whether co-worker presenteeism events occurred in the period of project delay) on the relationship between event strength and promotion focus.

#### Regulatory Focus Theory

The hedonic principle, which emphasizes approaching pleasure and avoiding pain, has become the basic motivational assumption of many psychological theories. In itself, however, the principle does not explain the different ways that it operates. Self-regulation, for example, is essential for adaptation because people need to adjust their cognition and action in the process of pursuing goals within various complex environments (Baumeister et al., 1993). Higgins (1997) thus went beyond the hedonic principle to put forward the RFT, which provides a clear answer to the operation of the principle. RFT distinguishes the type of self-regulation focused on promotion (accomplishments and aspirations) from that focused on prevention (safety and responsibilities; Higgins, 1997). When people are driven by goals of promotion, they will scrutinize their surroundings for information related to the pursuit of success, but when people are driven by goals of prevention, they will focus on information related to the avoidance of failure, and their subsequent behavior will correspond to this specific self-regulatory focus (Lockwood et al., 2002).

Kark and Van Dijk (2007) further divided individual regulatory focus into chronic regulatory focus and situational regulatory focus. Chronic regulatory focus refers to a relatively stable individual trait that is gradually formed during the growth process of an individual. Situational regulatory focus refers to the relatively more variable individual characteristics that are stimulated with the change of the contextual environment.

Therefore, regulatory focus is not only influenced by individuals’ personality (Wallace and Chen, 2006) but also evoked by environmental cues (Johnson et al., 2015). We assert that a co-worker presenteeism event can provide such a situational cue to arouse regulatory focus in employees. Then, the literature has long presented regulatory focus as a proximal motivational antecedent of work-related outcomes (Lanaj et al., 2012), so this study intends to use regulatory focus as an antecedent variable for innovative work behavior.

From the perspectives of EST and RFT, this study explores the effect of the strength of co-worker presenteeism events on employees’ innovative behavior. In addition, the indirect effects of regulatory focus in this relationship are analyzed and discussed. Finally, the boundary condition of the timing of co-worker presenteeism events on the relationship between event strength and employees’ regulatory focus is explored.

### Theoretical Model and Hypotheses

**Figure 1:**
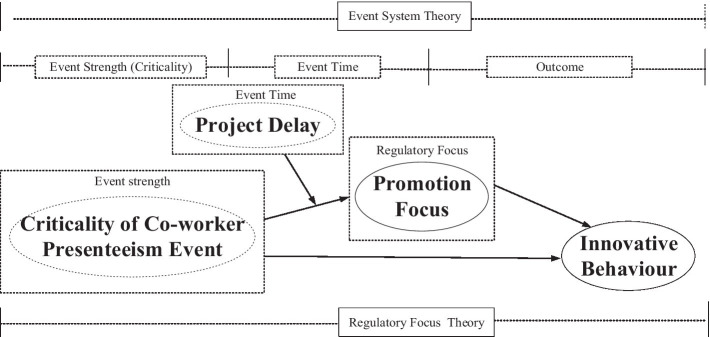
Research model.

Drawing on EST and RFT as the theoretical bases, we present our research model in Figure 1.

#### Effect of Criticality of Co-worker Presenteeism Events

Event criticality reflects ‘the degree to which an event is important, essential, or a priority’ to an entity (Morgeson and DeRue, 2006). It is not surprising that employees choose to attend work when experiencing suboptimal health under the 996 work regime of IT enterprises during and in the aftermath of the COVID-19 pandemic. Presenteeism is an adaptive job behavior that aims to balance health constraints and job demands, generally when experiencing noncontagious and common health problems (Karanika-Murray and Biron, 2019). It not only affects the physical and mental health and working relationships of employees but also has an impact on the productivity of an organization (Ruhle et al., 2020; Zhang et al., 2020).

The EST predicts that the more critical the event experienced by an entity, the more changes will be induced and the more attention and action will occur in response (Morgeson et al., 2015). We argue that a more critical co-worker presenteeism event will provide a greater incentive for innovative behavior. Furthermore, there are some circumstances under which going to work with an illness can be seen as making an effort to contribute and as a manifestation of organizational citizenship behavior (Johns, 2010; Miraglia and Johns, 2016). Some studies have shown that the altruistic behavior and interpersonal coordination found in organizational citizenship behavior are conducive to the formation of an atmosphere of innovation in a work team, which provides a foundation for increased individual innovation performance (Tang, 2005; Xu et al., 2016). Based on the integration of the above arguments, we hypothesize the following:

H1: Criticality of co-worker presenteeism events has a positive effect on innovative behavior.

#### Indirect Effect of Promotion Focus

Regulatory focus is not only influenced by personality (Wallace and Chen, 2006) but also evoked by situational cues (Johnson et al., 2015). The highly influential social learning theory proposes that individuals are likely to learn knowledge and norms by observing the behavior of others (Bandura, 1977). Observing the behavior of a co-worker is therefore an important form of situational stimulation for a specific self-regulatory focus among employees (Kim et al., 2021). Along these lines, we argue that a co-worker presenteeism event can provide such a situational cue to arouse regulatory focus in employees.

Scholars have conducted research on the regulatory mechanism during times of crisis. Markovits et al. (2014) argued that an economic crisis may encourage employees to pay more attention to the prevention rather than the promotion orientation and to use prevention as a strategy to cope with threatening circumstances, on the grounds that the economic crisis may reduce the chances of job promotion and success. However, the situation for the IT professionals in the present study is the opposite to that of an economic crisis, as the rapid development of digitalised industries, such as those involved in the provision of telecommuting, online medical care, online education, and online fresh food shopping, has been spurred by the COVID-19 pandemic. As the pandemic has provided space for the expansion of the related IT industries, we assert that co-worker presenteeism events will trigger promotion focus rather than prevention focus among IT professionals.

The basic relationship between the strength of co-worker presenteeism events and promotion focus has been clarified above, and we can also reveal the specific mechanism of inducing promotion focus through motivation-related theories. Any behavior and intention may stem from different motives, some from altruism and others from egoism (Ma et al., 2015; Lee et al., 2019), and promotion focus, like most human behavior and intentions, is caused by multiple motivations. The key point to distinguishing altruistic motivation from egoistic motivation is whether the ultimate purpose is self-serving (Batson, 1987). Existing studies on dispositional antecedents of promotion focus support this statement, such as altruistic-oriented conscientiousness and egoistic-oriented learning goal orientation (Gorman et al., 2012; Lanaj et al., 2012). Thus, the two coexistence mechanisms for inducing promotion focus will be elaborated separately from the aspects of altruism and egoism in the following.

The mechanism of altruistic perspective, mainly combined with the theory of social exchange, regards the co-worker presenteeism event as a kind of helping behavior, especially in background of collectivism culture in East Asia (Moorman and Blakely, 1995; Alabak et al., 2016). In return, individuals will do their best to do things for colleagues and organization, to pursue better self-growth. Indeed, this contention is further strengthened by social exchange theory, which proposes that individual behavior is intended for the mutually beneficial exchange of resources, with the core attributes of this exchange being self-interest and interdependence (Emerson, 1976; Lawler and Thye, 1999). Presenteeism is an adaptive behavior when it involves noncontagious and common health conditions (Karanika-Murray and Biron, 2019). Caverley et al. (2007) found that the primary reason for employees choosing to engage in presenteeism was the fear that their colleagues would have to take on additional job responsibilities if they were absent. To the extent that the strength of co-worker presenteeism events represents an atmosphere of co-workers helping each other, it may elicit a cognitive focus on accomplishment and growth rather than on duty and obligation. Thus, we argue that as the criticality of co-worker presenteeism increases, employees are more likely to be promotion oriented.

There is also an egoistic perspective in the inducing mechanism of promotion focus besides the altruistic perspective. People tend to take the initiative to seize all opportunities to seek better development in a fierce workplace environment. When colleagues come to work with illness, their work efficiency may be affected and employees may take advantage of this opportunity to show themselves and gain a better competitive advantage. The literature also shows that individuals with high egoistic values are more inclined to receive information that promotes focus orientation and adopt related behavior (Lagomarsino et al., 2020). Therefore, we assert that as the criticality of co-worker presenteeism increases, individuals are apt to be promotion oriented.

The literature has long presented regulatory focus as a proximal motivational antecedent of work-related outcomes (Lanaj et al., 2012). In the initial stages of research into regulatory focus and innovative performance, Friedman and Förster (2001) argued that compared to the perseverant processing method induced by prevention cues, the explorative processing method induced by promotion cues would facilitate creativity. Indeed, according to Baas et al. (2008), the promotion focus elicits widespread attention and facilitates the conceptual acquisition of mental representations with lower prior accessibility. Promotion-oriented employees are more inclined to adopt an open attitude to change and focus on exploratory behavior, whereas prevention-oriented employees are more focused on conservative behavior and more inclined toward stability (Neubert et al., 2008). This suggests that a promotion orientation may engender innovative behaviors in employees.

Building on the integration of the above arguments, it is plausible that a promotion focus might act as a mediator in the relationship between the criticality of co-worker presenteeism events and innovative behavior. Therefore, we propose the following hypothesis:

H2: Promotion focus has an indirect effect on the relationship between the criticality of co-worker presenteeism events and innovative behavior.

#### Moderating Role of Event Timing

Many companies must manage a portfolio of product development projects with a limited pool of resources. The competition between projects for the use of specific resources at specific times often results in project delays (Browning and Yassine, 2015), especially in IT enterprises (Haider and Kayani, 2020). With IT companies facing pressure to cope with the dynamic changes in market demand during and in the aftermath of the COVID-19 pandemic, they are highly likely to experience project delays.

Events have temporal characteristics that distinguish them from the constant features of a work environment. The timing of an event experienced by an entity can play a vital role in determining the impact of the event. The EST suggests that event timing moderates the relationship between event strength and outcome variables. Events that occur in time periods that match the development stage of the entity are more likely to trigger responses and generate change (Morgeson et al., 2015).

Based on the more severe work pressure faced by employees when the project they are working on is delayed, we argue that a co-worker presenteeism event occurring during a project postponement period is more likely to trigger a promotion focus than one occurring outside of a project postponement period. Thus, we hypothesize the following:

H3: The timing of co-worker presenteeism events moderates the relationship between the criticality of co-worker presenteeism events and promotion focus. Specifically, the effect is more significant when a co-worker presenteeism event occurs during a time of project delay.

## Materials And Methods

### Participants

With the administration of offline questionnaires not being possible during the COVID-19 pandemic, we conducted an online survey on the sojump.com platform to collect data for testing our research model. The data were collected from employees of Chinese IT companies. Participation in the study was voluntary, confidential, and anonymous. Upon completion of the questionnaire, each participant was given an electronic red envelope reward.

A total of 430 questionnaires were collected, and 374 questionnaires were obtained after deleting those with a total response time of less than 50 s and with the same number selected from beginning to end. Before proceeding with the statistical analyses, we identified multivariate outliers using Mahalanobis distance (1936) and verified the normality of the data. A multivariate outlier analysis was carried out according to the method of Mahalanobis distance (1936), and the results showed that two samples were outliers, so these two outliers were eliminated, and finally 374 valid samples were obtained. Then, scholars suggested that the values of skewness and kurtosis between −1 and +1 are acceptable for most psychometric purposes (Hair et al., 2009; George and Mallery, 2019). In the present study, the skewness and kurtosis values of the variables in the model fulfilled the criteria, indicating that the data were normally distributed. Prior research has indicated that demographic variables, such as gender, age, work experience, education and job category, are likely to be associated with innovative behavior (e.g., Zhang and Bartol, 2010; Zhang et al., 2016). Hence, consistent with previous studies, we controlled for these variables in our data analyses. After data cleaning, the sample comprised 374 employees, of which 67.91% were men and 32.09% were women. Most of the participants were between 20 and 45 years old: specifically, 1.87% (7) were aged below 21 years, 35.03% (131) were aged 21–25, 37.97% (142) were aged 26–30, 16.84% (63) were aged 31–35, 4.81% (18) were aged 36–40, 2.67% (10) were aged 41–45 and 0.80% (3) were 46 or older. Concerning education background, 27.81% of the respondents had a Master’s degree or above, 53.74% had a Bachelor’s degree and 18.45% had completed junior college. In terms of work experience, 17.91% (67) of the participants had 1 year or less, 32.09% (120) had 1–3 years, 22.99% (86) had 4–6 years, 14.17% (53) had 7–9 years and 12.83% (48) had 10 years or more. Based on the criteria used by major IT companies, the job categories of the participants were products (14.97%), technology (48.66%), operations (11.50%), marketing (5.61%), design (3.74%), administration (7.49%) and others (8.02%).

### Measures

All 18 items used to measure the latent variables were adapted from existing validated scales to fit the context of this study. All items were measured on a 7-point Likert scale ranging from 1 (*strongly disagree*) to 7 (*strongly agree*). The questionnaire was translated into Chinese using a back-translation procedure (Brislin, 1970). Considering the cultural adaptability of the measurement tools, we also referred to the corresponding measurement instruments of other papers with Chinese samples in addition to the translation and back-translation to adapt instruments. The good reliability and validity of those instruments have been well confirmed in Chinese populations. Three professors in the field of organizational behavior were asked to check the content of the items, and six graduate students employed in the IT industry were asked to complete the survey to check its clarity. This ensured that the participants would be able to understand the items clearly.

Innovative behavior was assessed using Scott and Bruce’s (1994) 6-item measure. The respondents were asked to rate the extent to which they engaged in certain behaviors (e.g., ‘I search out new technologies, processes, techniques, and/or product ideas’, ‘I generate creative ideas’ and ‘I develop adequate plans and schedules for the implementation of new ideas’) on a 7-point scale ranging from 1 (*strongly disagree*) to 7 (*strongly agree*). Cronbach’s alpha for this scale in this study was 0.93, showing good reliability.

Promotion focus was assessed with the 9-item measure of Lockwood et al. (2002). The respondents were asked to rate the extent to which they agreed with a number of statements (e.g., ‘I frequently imagine how I will achieve my hopes and aspirations’, ‘I typically focus on the success I hope to achieve in future’, ‘I see myself as someone who is primarily striving to reach my “ideal self” – to fulfill my hopes, wishes, and aspirations’ and ‘Overall, I am more oriented toward achieving success than preventing failure’) on a 7-point scale from 1 (*strongly disagree*) to 7 (*strongly agree*). Cronbach’s alpha for this scale in this study was 0.95, showing good reliability.

Criticality of co-worker presenteeism event used a 3-item event disruption scale developed by Morgeson and DeRue (2006) and translated into Chinese by Liu and Liu (2017). The respondents were asked the following screening question before the three items were presented as follows:

A co-worker presenteeism event refers to the behavior of a colleague participating in work in a state of ill-health (having a backache, cold, mental health issue, etc.). If a colleague is in the above situation, please continue to fill in the questionnaire. If the above situation does not exist, please exit the questionnaire (screening question).

The respondents were then asked to rate the extent to which they agreed with three statements on a 7-point scale from 1 (*strongly disagree*) to 7 (*strongly agree*). Two sample items were ‘The co-worker presenteeism event is critical for the long-term success of the team’ and ‘The co-worker presenteeism event is important for the team’. Cronbach’s alpha for criticality of co-worker presenteeism event in this study was 0.87, showing good reliability.

The timing of the co-worker presenteeism event was measured with a single item asking ‘whether the recent co-worker presenteeism event occurred in a period of project delay’, for which respondents could select *yes* or *no* (coded as 1 and 0, respectively).

### Data Analysis

Data preparation and all statistical analyses, including confirmatory factor analysis (CFA), common method variance (CMV), descriptive statistics, and hypotheses testing, were conducted with SPSS (version 23) and Amos (version 20).

The analysis had three steps. First, CFA was conducted to assess the discriminant validity of the core variables, and the CMV was examined. Second, the descriptive statistics and correlations between key variables were analyzed. Third, the postulated hypotheses were tested.

Measures of global fit were checked during model testing. The criteria used to evaluate reasonable global fit were chi-square minimum degrees of freedom (*χ*^2^/*df*) <5 (Yang et al., 2016), root-mean-square error of approximation (RMSEA) <0.08 (Hu and Bentler, 1999), nonnormed fit index (NNFI) and comparative fit index (CFI) ≥0.90 (Cheung and Rensvold, 2002).

## Results

### Discriminant Validity and Common Method Variance

Using Amos (version 20), we tested the discriminant validity with CFA. The CFA results indicate that our proposed three-factor model (criticality of co-worker presenteeism event, promotion focus and innovative behavior) yielded a better fit than alternative models (Model 1: *χ*^2^/*df* = 3.154, RMSEA = 0.076, CFI = 0.949, NNFI = 0.940; Model 1 in order to test the discriminant validity between criticality of co-worker presenteeism event, promotion focus and innovative behavior; Model 2 in order to differentiate co-worker presenteeism event + promotion focus and innovative behavior; Model 3 in order to test whether above variables belong to one factor; Model 4 followed the suggestion of Podsakoff et al. (2003), and the unmeasured latent methods factor was applied, in order to test CMV; see Table 1). Therefore, the measures of the three core variables in this study captured the distinct constructs.

**Table 1:** Results of confirmatory factor analysis (*n* = 374).

Model	*χ*^2^	*df*	*χ*^2^/*df*	RMSEA	CFI	NNFI	Δ*χ*^2^	Δ*df*
Model 1 (three factors: CP, PF and IB)	411.211	130	3.163	0.076	0.946	0.937		
Model 2 (two factors: CP + PF and IB)	930.562	134	6.944	0.126	0.847	0.826	519.351^***^	4
Model 3 (one factor: CP + PF + IB)	1616.286	135	11.972	0.172	0.716	0.678	1205.075^***^	5
Model 4 (unmeasured latent methods factor)	277.105	112	2.474	0.063	0.968	0.957	134.106^***^	18

*CP, criticality of co-worker presenteeism event; PF, promotion focus; IB, innovative behavior.*

*** *p < 0.001*.

Common method variance was a potential problem in this study given of the use of a self-report questionnaire from a single source. A CFA was used to examine the issue. Following the suggestion of Podsakoff et al. (2003), the unmeasured latent methods factor was also applied. A latent method factor was constructed based on the original three-factor structure (i.e., the items for criticality of co-worker presenteeism event, promotion focus and innovative behavior loading on their respective constructs). The latent methods factor was uncorrelated with other factors, and all of the items were loaded on this latent methods factor.

A comparison of the unmeasured latent methods factor model and the theoretical model indicated a slight change of chi-square value, Δ*χ*^2^(18) = 135.359, *p* < 0.001 (see Table 1). Chi-square values are easily impacted by sample size, especially when the sample size is larger than 200 (Cheung and Rensvold, 2002; Zhu and Zhang, 2019). Therefore, researchers have suggested examining the NNFI for model choice, with a change of NNFI of less than 0.05 indicating that adding the unmeasured latent methods factor does not significantly promote the theoretical model (Little, 1997; Zhu and Zhang, 2019). Given that the sample size in this study was 374, we followed this procedure and found that NNFI increased by 0.02 when the latent methods factor was included. Therefore, adding a latent methods factor did not significantly improve the model, and we concluded that CMV did not have a significant impact on the results.

### Descriptive Statistics

The means, standard deviations and correlation matrices of the key variables are presented in Table 2. Criticality of co-worker presenteeism event was positively correlated with innovative behavior (*r* = 0.40, *p* < 0.01) and with promotion focus (*r* = 0.46, *p* < 0.01). Promotion focus was positively correlated with innovative behavior (*r* = 0.66, *p* < 0.01). The correlation results were in accordance with our hypotheses and indicated suitability for further hypothesis testing.

**Table 2:** Means, standard deviations and correlations for latent variables.

Variable	*M*	*SD*	CP	PF
Criticality of co-worker presenteeism event (CP)	4.99	1.11		
Promotion focus (PF)	5.47	0.95	0.46^**^	
Innovative behavior (IB)	5.42	0.96	0.40^**^	0.66^**^

** *p < 0.01*.

### Hypothesis Testing

H1 predicts that criticality of co-worker presenteeism event is positively related to employees’ innovative behavior. To test the direct effect, we controlled demographic variables. As presented in Table 3, M2 shows that criticality of co-worker presenteeism event has a significant effect on employees’ innovative behavior (*β* = 0.35, *p* < 0.001). Therefore, H1 is supported.

**Table 3:** Hierarchical regression analysis.

Predictors	PF		IB	
	M1	M2	M3	M4
*Control variables*				
Job category	0.04	−0.00	−0.02	−0.03
Gender	0.02	0.01	−0.02	0.00
Education level	0.02	0.09	0.07	0.08
Age	−0.02	0.16^*^	0.17^**^	0.17^**^
Work experience	0.05	−0.05	−0.09	−0.09
*Independent variable*				
CP	0.39^***^	0.35^***^		0.11^**^
*Mediator*				
PF			0.68^***^	0.62^***^
*F*	16.62^***^	13.75^***^	52.88^***^	47.42^***^
*R*^2^	0.21	0.18	0.46	0.48

*CP, criticality of co-worker presenteeism event; PF, promotion focus; IB, innovative behavior.*

* *p < 0.05;*

** *p < 0.01;*

*** *p < 0.001*.

H2 asserts that promotion focus would mediate the relationship between criticality of co-worker presenteeism event and employees’ innovative behavior. We followed mediation testing procedure from Baron and Kenny (1986) to verify H2. As given in Table 3, M1 shows that the effect of criticality of co-worker presenteeism event on promotion focus is significant (*β* = 0.39, *p* < 0.001), M3 indicates promotion focus has a significant effect on innovative behavior (*β* = 0.68, *p* < 0.001) and M4 indicates that after joining promotion focus, the effect of criticality of co-worker presenteeism event on employees’ innovative behavior is decreased, but still significant (*β* = 0.11, *p* < 0.01). *R*^2^ was 0.46 (*p* < 0.001). Thus, H2 is supported.

**Figure 2:**
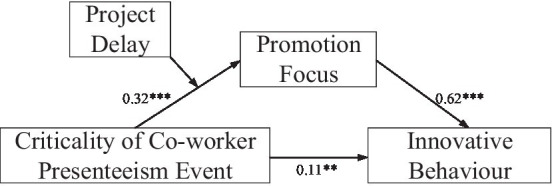
Results of research model by using model 7 of the PROCESS. ^**^p < 0.01; ^***^p < 0.001.

Then, following the procedure of Baron and Kenny (1986), we used model 7 of the PROCESS macro in SPSS to test H3 and the whole research model (see Figure 2). Our results show that criticality of co-worker presenteeism event was significantly and positively correlated with employees’ innovative behavior (*β* = 0.11, *p* < 0.01). There was a significant positive association between criticality of co-worker presenteeism event and promotion focus (*β* = 0.32, *p* < 0.001). Promotion focus was positively associated with innovative behavior (*β* = 0.62, *p* < 0.001). As expected, results confirmed that the interaction between criticality of co-worker presenteeism event and the timing of co-worker presenteeism events (co-worker presenteeism events occurs during a time of project delay or not) on promotion focus was significant (*β* = 0.18, *p* < 0.05). The results of model 7 further showed that the mediating effect of promotion focus in the relationship between criticality of co-worker presenteeism event and employees’ innovative behavior during a project postponement period (effect = 0.31, 95% CIs [0.22, 0.39]) was stronger than one occurring outside of a project postponement period (effect = 0.20, 95% CIs [0.11, 0.29]). H3 is therefore well supported. All results are marked in Figure 2.

## Discussion

Drawing on the EST and RFT, this study constructed an impact mechanism model to investigate the relationship between the strength of co-worker presenteeism events and innovative behavior among IT professionals against the background of 996 work regime. The direct effect was tested alongside the indirect effect of promotion focus and the moderating effect of event time in this relationship. An online survey was administered to 374 IT professionals in China. The results showed a positive direct relationship between criticality of co-worker presenteeism event and innovative behavior and an indirect effect of promotion focus in this relationship. Furthermore, the timing of a co-worker presenteeism event during a project delay moderated the relationship between criticality of co-worker presenteeism event and promotion focus, with the effect stronger when co-worker presenteeism events occurred during project delays. These findings have important theoretical and practical implications.

### Theoretical Implications

The findings make several contributions to the literature on presenteeism and innovative behavior. First, most studies on the consequences of presenteeism have focused on its negative outcomes, arguing that it is bad for productivity. Relatively fewer studies have examined the positive effects of presenteeism, although several have described presenteeism as a kind of adaptive (Karanika-Murray and Biron, 2019) or organizational citizenship behavior (Miraglia and Johns, 2016) that is beneficial to individual innovation performance (Xu et al., 2016). Based on EST, this study expands the scope of research into the positive impact of presenteeism by suggesting that it can facilitate individual innovative behavior in co-workers.

Second, most studies of presenteeism have focused on individual effects, positive and negative, but few have attended to interpersonal effects, such as whether and how presenteeism on the part of a co-worker can affect the emotions, cognition or behavior of an employee (Luksyte et al., 2015). With the aid of EST (Morgeson et al., 2015), this study extends presenteeism to include co-worker presenteeism, regarded as an event, and explores the influence of the criticality of presenteeism events on the innovative behavior of colleagues from an interpersonal perspective.

Third, studies of the mediation mechanism between co-worker presenteeism and its outcomes have mostly concentrated on discrete emotional responses (Luksyte et al., 2015), which are relatively transient. However, the acts of colleagues can also arouse responses in some relatively stable traits, such as individual self-regulation preference. According to the EST and RFT, each person has a different regulatory focus for coping and responds differently to events occurring at different times. Furthermore, as mentioned in H2 above, the way in which the promotion focus works, in addition to the explanation from the help behavior, may also include taking advantage of the disadvantaged situation of colleagues or other possible deleterious effects. This study thus expands the research into the mediation mechanism between co-worker presenteeism and employees’ innovative behavior from the perspective of self-regulation. In addition, considering the background of the 996 work regime of IT companies during and in the aftermath of COVID-19 pandemic, we discuss the event time boundary conditions for the relationship between criticality of co-worker presenteeism events and a promotion-oriented regulatory focus, specifically in relation to the project delays that are often experienced by IT enterprises. This study thus enriches the theoretical understanding of the mechanism of the positive effect of presenteeism, and it is also an extension of the EST due to the increased mediating factor combined with the RFT.

Finally, research on the antecedents of innovative behavior, such as the attributes of the work, individual personality traits or such situational factors as leadership style and organizational climate, has mostly focused on the stable characteristics of the entities under study and has rarely explored the event-related antecedents. This study thus deepens the innovative behavior antecedents research by adding the perspective of event-related factors.

### Managerial Implications

Our findings have two valuable practical implications. First, our results indicated that criticality of co-worker presenteeism events had a direct effect on innovative behavior and an indirect effect *via* promotion focus by eliciting widespread attention and the adoption of a more open attitude to changes and exploratory behaviors. Organizations should be aware of such effects, especially against a macro background of the coexistence of crisis and opportunity as exists during COVID-19, in appropriately exerting a certain degree of work pressure and striving to increase the promotion focus of employees, thereby facilitating their innovative job behavior. In particular, since the negative effects of presenteeism mentioned in the existing studies do exist, although the conclusion of this study is that co-worker presenteeism events are conducive to employees’ innovative behavior, it does not mean that organization managers should openly advocate presenteeism, but we should treat the phenomenon of presenteeism more objectively, and not necessarily resist it all at once. Instead, we should formulate corresponding strategies according to the actual needs of the organization. Second, the results shed light on the moderating role of the timing of co-worker presenteeism events by showing that the relationship between criticality of co-worker presenteeism events and promotion focus was stronger when co-worker presenteeism events occurred during project delays. Hence, organizations should take steps to improve employees’ promotion focus during periods of project delay.

### Limitations and Future Research

There are several limitations to this study. First, the cross-sectional design limits the ability to make causal inference about the proposed relationships. Thus, scholars may consider multi-wave design or dynamic model to examine the corresponding hypotheses in future, and adopt methods, such as longitudinal research to improve the validity of research conclusions. In addition, future research can expand the sample size and increase the representativeness of the sample.

Second, our study used a self-report questionnaire, which can lead to a degree of common method bias (Podsakoff et al., 2003). Although testing of the unmeasured latent methods factor indicated that common method bias did not seriously affect our results, the use of time-lagged, longitudinal and multi-source data would be beneficial in future research. Specifically, two waves of data collection are suggested, with subordinates asked to complete the questionnaire on the criticality of co-worker presenteeism events and promotion focus at Time 1 and supervisors asked to evaluate their subordinates’ innovative behavior at Time 2.

Third, whereas this study examined the regulatory focus mechanism of the positive effect of co-worker presenteeism events on innovative behavior, however, the discussion on the possible deleterious effects of promotion focus is not sufficient, and further empirical research and theoretical interpretation can be done in future. We also encourage scholars to test other underlying mechanisms, such as regulatory modes (Li et al., 2018) and emotional mechanisms, that may explain the possible positive effects of co-worker presenteeism events.

Fourth, this study treated the criticality of a co-worker presenteeism event as an important antecedent of employees’ innovative behavior. To gain a deeper understanding of the influence of colleague presenteeism events grounded in EST, we suggest investigation of other event-related attributes, such as disruption and novelty, of co-worker presenteeism (Morgeson et al., 2015). In addition, we explored the boundary condition of the timing of co-worker presenteeism events on the relationship between event strength and employees’ regulatory focus. Based on EST, event space (origin, spatial dispersion, etc.) might also have individual or collective effects on the entity. For a deeper understanding of the effects of co-worker presenteeism events, the possible boundary conditions of event space should be further explored.

Finally, our study was performed in a single country, China, against the background of the 996 work regime of the IT industry. Because cultural differences have been considered important with respect to innovation (Rosenbusch et al., 2011), they may influence the relationships between co-worker presenteeism events and employees’ innovative behavior. We therefore encourage future research in other cultural contexts and cross-cultural research.

## Data Availability Statement

The raw data supporting the conclusions of this article will be made available by the authors, without undue reservation.

## Ethics Statement

The studies involving human participants were reviewed and approved by the Review Board of the First Affiliated Hospital of Xiamen University, China (KYX2016007). The patients/participants provided their written informed consent to participate in this study. Written informed consent was obtained from the individual(s) for the publication of any potentially identifiable images or data included in this article.

## Author Contributions

TY, RL, and JD originated and designed the study and contributed to the statistical analysis, interpretation of the results, and revision of the manuscript. RL and TY wrote the paper. All authors were involved in editing, reviewing, and providing feedback for this manuscript and have given approval of the final version to be published.

### Conflict of Interest

The authors declare that the research was conducted in the absence of any commercial or financial relationships that could be construed as a potential conflict of interest.
